# Recurrent gastric ulcer and cholangitis caused by ectopic drainage of the bile duct into the stomach

**DOI:** 10.3205/iprs000114

**Published:** 2017-08-10

**Authors:** Katrin Bauer, Christof Keller

**Affiliations:** 1 Department for General, Visceral, Vascular, Thoracic and Pediatric Surgery of the Kempten Clinic, Kempten, Germany

**Keywords:** ectopic orifice of the bile duct

## Abstract

We report a case of recurrent gastritis with pyloric stenosis and cholangitis due to a rare variation in bile duct anatomy. A 72-year-old female patient showed recurrent gastral ulcers and biliary colic with cholangitis caused by gallstones in the main bile duct with an ectopic orifice in the prepyloric region and concurrent inflammatory pyloric stenosis. After temporarily successful endoscopic treatment with stenting and pyloric dilatation, the patient suffered from recurrent cholangitis. Finally, the abnormal biliary anatomy required surgical treatment with biliodigestive anastomosis.

## Zusammenfassung

Wir berichten über eine 72-jährige Patientin, die sich mit rezidivierenden Magenulcera, Gallenkoliken und einer Pylorusstenose bei uns vorstellte. Als Ursache wurde eine ektopische Mündung des Gallengangs in den präpylorischen Magen diagnostiziert. Zusätzlich bestanden eine Pylorusstenose und rezidivierende Cholangitiden mit Leberabszessen. Nach vorübergehender Besserung der Symptome durch eine endoskopische Stenteinlage war letztlich die operative Anlage einer biliodigestiven Anastomose notwendig.

## Introduction

Vesalius in 1543 was the first to report on an aberrant bile duct. Since then, numerous reports based on autopsy studies, radiologic imaging, and surgical specimens have elucidated the enormous variability of the biliary system. Besides the developmental duplication in different variations or even a complete lack of a normal bile duct, the biliary system can also drain into atypical regions of the duodenum and very rarely the stomach.

## Case description

A 72-year-old female patient with a history of obesity (BMI 37 kg/m^2^), gastroesophageal reflux disease and multiple gastroduodenal ulcers presented to our department with right upper quadrant pain, fever, and clinical signs of biliary colic.

**Figure 1:**
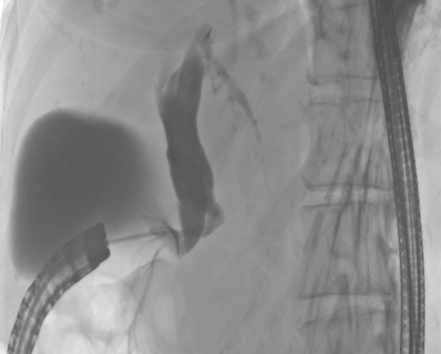
ERCP with gallbladder and widened bile duct

**Figure 2:**
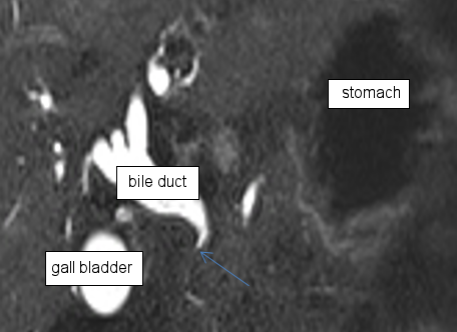
MRI with widened bile duct and the ectopic orifice into the pyloric region

Abdominal ultrasound showed a distinct intra- and extrahepatic widening of the biliary tract and 2 prepapillary gallstones in the main bile duct. Blood tests showed increased values for GOT, GPT, γ-GT, AP, and CRP. The values for bilirubin and lipase were normal. Esophagogastroduodenoscopy (EGD) revealed a stenosis of the pylorus and an erosive duodenitis with a postpyloric diverticulum and concurrent antral gastritis. Furthermore there was an ectopic orifice with biliary secretion in the prepyloric stomach. A normal papilla in the duodenum was not found. Endoscopic retrograde cholangiopancreatography (ERCP) with selective intubation of the prepyloric orifice showed a very short and widened biliary tract (Figure 1 (Fig. 1)). Choledocholithiasis could not be confirmed. A magnetic resonance cholangiopancreatography (MRCP) scan confirmed the ectopical drainage of the lightly widened common bile duct into the prepyloric region (Figure 2 (Fig. 2)). Enlarged lymph nodes in the hepatoduodenal region were present. 

The pyloric stenosis was endoscopically dilated and a removable stent was placed into the pyloric bile duct orifice. Endoscopic control after 8 weeks showed healing of the gastritis under therapy with pantoprazole. The stent was removed. Ultrasound showed persisting dilatation of the bile duct (16 mm), γ-GT, AP, GOT, and GPT remained slightly elevated. The patient was asymptomatic. 

After 4 years of doing well, the patient was readmitted with epigastric pain and bilious vomiting. The blood tests showed increased values for leucocytes, CRP, GOT, GPT, γ-GT, and AP consistent with cholangitis. The values for bilirubin and lipase were regular. ERCP, CT, and MRCP confirmed the formerly described anatomic abnormalities. CT scan raised suspicion for hepatic abscess formation. Gallstones could again not be extracted during ERCP.

## Therapy

Due to the recurrent cholangitis and suspected liver abscess formation the patient was started on ciprofloxacin and metronidazol, five days later explorative laparotomy was performed. The stone-filled gallbladder was removed first. A long cystic duct could be followed to the prepyloric region. There was chronic inflammation of the hepatoduodenal ligament with mild lymphadenopathy, a lymph node, and a tumor of the third hepatic segment were removed for pathological examination. Cryosection showed inflammatory changes without evidence for malignancy. The pancreatic head and the peripancreatic region were explored without finding a tumor or further abnormalities. Decision for the creation of a biliodigestive anastomosis was made. Therefore the pyloric region with the ectopic orifice of the bile duct was resected and a retrocolic end-to-side Roux-en-Y biliodigestive anastomosis was created. The patient recovered well, follow-up after 3 months was unremarkable.

**Figure 3:**
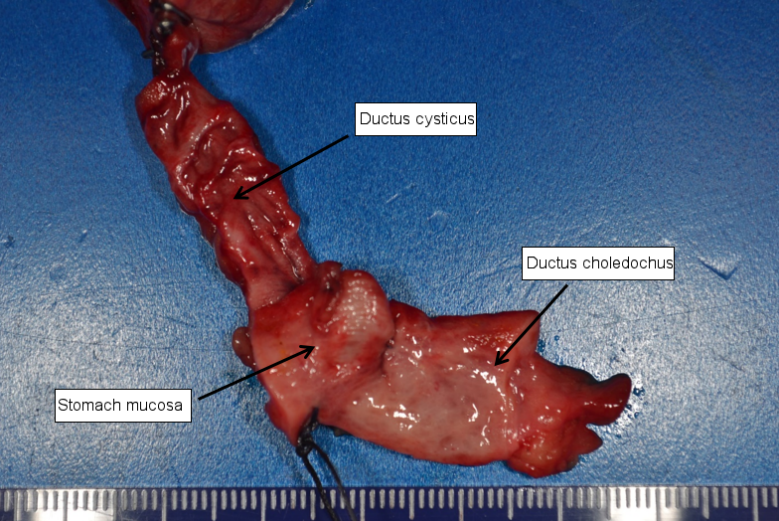
Ductus cysticus and choledochus with their ectopic orifices into the prepyloric stomach

Histopathological examination showed chronic inflammatory changes of the gallbladder, the resected bile duct and the removed lymph node. The hepatic specimen was consistent with liver abscess. Interestingly, there was no confluence of the cystic duct with the bile duct and a separate orifice for the cystic duct in the prepyloric region (Figure 3 (Fig. 3)).

## Diagnosis

Cholangitis with liver abscess formation caused by choledocholithiasis complicated by an ectopic orifice of the bile and cystic duct into the prepyloric stomach.

## Discussion

The majority of the described cases of ectopic biliary orifice is caused by developmental abnormalities in the biliary system, which arises from the ventral mesenterium in the fifth embryonic week [1]. Furthermore, there are even rarer cases of secondary changes to the biliary system with fistula formation due to inflammation or malignancy. In most cases of developmental ectopic bile drainage, there is an orifice into the duodenum and only very rarely into the stomach [2], [3], [4]. As seen in the described case, an ectopic orifice of the bile duct into the pyloric region can be associated with different problems. Chronic gastritis and stenosis of the the pyloric region have been described as well as recurrent cholangitis. Bile stasis by unphysiological configuration of the bile duct and a purely developed or missing sphincter Oddi are considered as the main reasons for the symptomatology. Alterated bile flow through spincter dysfunction and angulated anatomy of the ducts can lead to stone formation and cholangitis 

## Conclusion

Ectopic biliary drainage in the stomach is a very rare condition, which can cause problems due to gastritis with ulcer formation and consequently pyloric stenosis. In addition, functionally and anatomically altered bile duct anatomy predisposes to cholangitis and stone formation. ERCP is the mainstay of treatment, but as seen in our case, reconstructive biliary surgery may be needed in some cases. 

## Notes

### Competing interests

The authors declare that they have no competing interests.
